# Influence of baking and frying conditions on acrylamide formation in various prepared bakery, snack, and fried products

**DOI:** 10.3389/fnut.2022.1011384

**Published:** 2022-11-21

**Authors:** Muhammad Mushtaq Ahmad, Tahir Mahmood Qureshi, Maham Mushtaq, Amjad Islam Aqib, Umair Mushtaq, Salam A. Ibrahim, Abdul Rehman, Muhammad Waheed Iqbal, Tabish Imran, Shahida Anusha Siddiqui, Anjum Javed, Sadaf Shamim, Muhammad Hamzah Saleem

**Affiliations:** ^1^Institute of Food Science and Nutrition, University of Sargodha, Sargodha, Pakistan; ^2^Department of Food Sciences, Cholistan University of Veterinary and Animal Sciences, Bahawalpur, Pakistan; ^3^National Institute of Food Science and Technology, University of Agriculture, Faisalabad, Pakistan; ^4^Department of Medicine, Cholistan University of Veterinary and Animal Sciences, Bahawalpur, Pakistan; ^5^Department of Pharmacy, Government College University, Faisalabad, Pakistan; ^6^Department of Food and Nutritional Sciences, North Carolina Agricultural and Technical State University, Greensboro, NC, United States; ^7^School of Food and Biological Engineering, Jiangsu University, Zhenjiang, Jiangsu, China; ^8^Technical University of Munich Campus Straubing for Biotechnology and Sustainability, Straubing, Germany; ^9^German Institute of Food Technologies (DIL e.V.), Quakenbrück, Germany; ^10^Wheat Research Institute, Ayub Agricultural Research Institute, Faisalabad, Pakistan; ^11^Office of Academic Research, Office of Vice President (VP) for Research and Graduate Studies, Qatar University, Doha, Qatar

**Keywords:** flour rheology, acrylamide concentrations, sensory evaluation, bakery, fried products

## Abstract

The core objective of the present study was to evaluate the influence of baking/frying times and temperatures on the formation of acrylamide in bakery, snack, and fried products such as biscuits, muffins, pizza, cakes, samosa, paratha rolls, nuggets, and potato cutlets during baking/frying at different times and temperature conditions. First of all, the raw material, especially flour, was tested for its proximate composition and rheological characteristics. The quantification of acrylamide produced during the processing of different products was carried out through the HPLC method. A sensory evaluation of these food samples was also carried out to find out the acceptability differences. The raw material was found to have good rheological properties and proximate composition. The results revealed that different times and temperature regimes influenced the formation of acrylamide in those products. Among the bakery products, the highest concentrations of acrylamide were observed in biscuits (126.52 μg/kg) followed by muffins (84.24 μg/kg), cake (71.21 μg/kg), and pizza (62.42 μg/kg). The higher contents of acrylamide were found in paratha roll (165.92 μg/kg) compared to samosa (100.43 μg/kg), whereas among snacks, potato cutlets (135.71 μg/kg) showed higher concentrations than nuggets (43.04 μg/kg). It was observed that baking or frying all the investigated products at higher temperatures produced slightly more acrylamide concentrations. The prepared products in the present study were also accepted sensorially by the panel of judges. So, it was concluded that baking or frying at higher temperatures resulted in higher concentrations of acrylamide compounds in different products in the present study.

## Introduction

Acrylamide (C_3_H_5_NO; 2-propenamide) is considered an odorless and crystalline solid compound having a low molecular weight (71.08 kDa) and is highly water soluble (1–3). Usually, it is formed in foods with high-carbohydrate contents, that is, breakfast cereal, bakery products, potato crisp, roast potatoes, and roasted coffee, as a result of cooking practices (4, 5). During such kind of cooking, the Maillard reaction is responsible for the formation of acrylamide compound as a result of the reaction between carbohydrates and amino acids, specifically asparagine’s (6). It has been reported that the major precursors resulting in the formation of acrylamide are reducing sugars (fructose, glucose, and maltose) and asparagine (7, 8), which are usually formed in foods prepared above 120°C and with low moisture (1). Other minor routes of acrylamide formation may include 2-propenal and acrylic acid in the presence of amino acids having nitrogen in their side chains, for instance, lysine, glutamine, and arginine or a β-proton in the amino acid adjacent to alanine (7, 9, 10).

It has been reported that acrylamide can increase the risk of tumors of the mammary glands, thyroid gland-follicular epithelium, central nervous system, colon, uterus, and clitoral gland in rats (11, 12). Moreover, acrylamide also has been reported to have negative impacts on public health, such as decreasing immune and blood systems (13–15). Acrylamide is classified as a probable carcinogen by the International Agency for Research on Cancer (16) and is also considered as a neurotoxin announced by (17, 18). Furthermore, neurotoxicity, developmental toxicity, adverse effects on male reproduction, and carcinogenicity were also reported to be identified as possible critical endpoints for AA toxicity from different animal studies (1).

The presence of acrylamide in foods attracted public alarm after its determination in heated starch-based food products to be used in routine by the Swedish National Food Administration (19). Several organizations started to ponder over the estimated health risk of acrylamide in food products (20). In addition, European Commission also started monitoring acrylamide levels in different processed foods (21). Similarly, to consider the adoption of possible risk management strategies, the European Commission also issued further recommendations to examine the presence of acrylamide in bakery products (croissants, doughnuts, pancakes, etc.), potato-based products (croquettes, potato casserole, potato/meat dishes, etc.), cereal products (rice and maize crackers, cereal snacks, etc.), as well as other foods (dried fruits, olives in brine, vegetable crisps, etc.) (22).

There might be great variability in cooking/processing practices at domestic, catering services, and industrial levels which directly influence the formation of variable quantities of acrylamide, ultimately impacting the health of consumers. Such kind of variability arises from variations in raw materials, recipes, and processing conditions. For example, in a previous study conducted in 208 Spanish households, it was revealed that there was great variability in acrylamide contents in fried potatoes (a simple process) (23). Similarly, variations in acrylamide contents were observed in different bakery products (24).

Different strategies have been reported to reduce the formation of acrylamide in different food products. Among those strategies, the use of suitable raw material, blanching, fermentation, time, and temperature combinations are crucial for minimizing the threats of more acrylamide formation in different food products (reviewed by (4)).

In Pakistan, different bakery and fried products are produced at catering services as well as industrial levels. The people working in the manufacturing section usually use oils and fats which is left over from previous days. They use such kind of poor quality material (oils and fats) which was used/heated several times over. Therefore, there might be the possibility of the formation of more and more acrylamide contents due to the intensive processing temperature of those products.

To have public awareness, there was a dire need to investigate the formation of acrylamide in different fried and bakery products prepared according to standardized recipes and procedures. So far, no comprehensive study has been published regarding the formation of acrylamide during the processing of different fried and bakery products which are locally manufactured and used by the people of Pakistan. Hence, the main objective and novelty of the present study were to investigate acrylamide contents in various bakery, snacks, and fried products which are locally used by the people of Pakistan.

## Materials and methods

In the present study, four bakery products (biscuits, cakes, muffins, and pizza) and four snacks/fried products (samosa, paratha, chicken nuggets, and potato cutlets) were produced in the cereal department of Ayub Agricultural Research Institute, Jhang road, Faisalabad. The above products were prepared according to standard recipes and procedures for each product (Tables 1–8). The treatment plan of the present is given in Table 9. Three batches of each product were prepared at different times and temperature conditions.

**TABLE 1:** Ingredients and procedure for preparation of pizza.

Ingredients	Measures	Procedure
Onion Garlic powder Oil Chicken boneless	2 no 1 Tbsp 250 g 2 cups	1. The oven was pre-heated at 200°C. 2. Onion, garlic powder, spices, and chicken were added in pan and fried.
Milk Yeast Eggs All-purpose flour Salt Sugar	2 cups 5 g 2 no 4 cups 5 points 20 gram	3. Yeast was added into milk and placed in proofer for 20 min. 4. After proofing milk, egg, salt and sugar were added in flour to make dough. 5. The dough was again placed in proofer for 20 min.
Tomato puree Black olives and spices Vanilla extract Mozzarella cheese Cheddar cheese	1 cup 50 g 1/2 teaspoon 250 gram 250 gram	6. Two replications were made and then the bread sheeting was done after 20 min. Then tomato paste, cheese, chicken, and olive were applied on sheet step by step and baked in oven at 190°C (T_1_) and 210°C (T_2_) for 20 min each.

**TABLE 2:** Ingredients and procedure for preparation of cake.

Ingredients	Measures	Procedure
Sugar Ghee (Kashmir-brand)	4 cups 4 cups	1. The oven was pre-heated at 180°C. 2. Ghee and sugar were mixed in a mixer for 30 min.
All-purpose flour Eggs Milk Vanilla extract	4 cups 4 no 2 cups 1/2 tsp	3. Add flour, eggs, vanilla extract and milk were added in mixer to mix for 15 min. 4. Two replications were made and then the mixtures were added in pans and baked at 170°C (T_1_) and 190°C (T_2_) for 20 min each.

**TABLE 3:** Ingredients and procedure for preparation of muffins.

Ingredients	Measures	Procedure
Sugar Ghee (Kashmir-brand)	4 cups 4 cups	1. The oven was pre-heated at 180°C. 2. Ghee and sugar were added in a mixer to mix for 30 min.
All-purpose flour Eggs Milk Vanilla extract	4 cups 4 no 2 cups 1/2 tsp	3. Flour, eggs, vanilla extract and milk were added in mixer to mix for 15 min. 4. Two replications were made and then the mixtures were added in muffin pans and baked at 170°C (T_1_) and 190°C (T_2_) for 20 min each.

**TABLE 4:** Ingredients and procedure for preparation of biscuits.

Ingredients	Measures	Procedure
Butter, softened Sugar Eggs Milk Vanilla extract	2, 1/2 cups 2 cups 2 no 1/4 cup 1–2 tsp	1. The oven was pre-heat at 190°C. 2. Cream butter and sugar were mixed in a large mixing bowl. Then eggs, milk and vanilla extract were added and mixed well.
All-purpose flour Baking powder Salt	8 cups 4 tsp 1 tsp	3. The flour, baking powder and salt were combined together. 4. The above dry mixture (step 3) was gradually added into creamy mixture (from step 2) and beating was done just until combined. 5. The mixture was developed into sheaths and cut according to the biscuit size with the help of moulds. 6. Baking was done at 170°C (T_1_) and 190°C (T_2_) for 20 min each.

**TABLE 5:** Ingredients and procedure for preparation of samosa.

Ingredients	Measures	Procedure
Potatoes	1/2 kilo 1 tsp 1/2 tsp Few	1. Firstly paste was made by mixing the boiled potato with dried pomegranate seeds, red chilies, and green chilies.
Dried pomegranate seeds Red chillies Green chillies		
Carom seeds All-purpose flour Oil	1/2 tsp 1/2 kilo 1/2 cup, 2 kilo	2. Then dough was made by kneading flour with carom seeds and 1/2 cup of oil. 3. The sheaths were developed from prepared dough in triangular form and filled the paste in these triangle shaped material and then frying was done in the cooking oil. In this way, samosas were prepared. Two treatments were given to samosas at 180°C for 4 (T_1_) and 5 min (T_2_) each.

**TABLE 6:** Ingredients and procedure for preparation of paratha roll.

Ingredients	Measures	Procedure
Boneless chicken Vinegar Soya sauce Salt Lemon juice	½ kilo 2 tsp + 2 tsp 1tsp Few points 1tsp	1. Firstly, boneless chicken with vinegar, soya sauce, salt, lemon juice were marinated, and then this marinated material was refrigerated overnight.
Garlic paste Onion Butter Carom seeds Black pepper White cumin seeds	2 tsp 1 small As needed 1/2 tsp 1 tsp 1 tsp	2. Garlic and onion were fried in butter and the mixture of step 1 was poured into this fried mixture. After this, carom seeds, black pepper, and white cumin seeds were also added.
Flour Salt	1/2 kilo Few points	3. The remaining (2 tsp) of vinegar were added into onion and cucumber placed them at separate place. 4. The parathas were prepared from flour dough and all the prepared material from step 1, 2 and 3 were added into prepared parathas and rolled. These parathas were fried at 180°C temperature for 4 min (T_1_) and 5 min (T_2_) each.

**TABLE 7:** Ingredients and procedure for preparation of potato cutlets.

Ingredients	Measures	Procedure
Boiled potatoes Red chilies Green chilies Coriander Turmeric powder Dried seeds of pomegranate Salt	1/2 kilo 1/2 tsp Few 1 tsp 1/2 tsp 1tsp Few pints	1. Firstly, paste was made by mixing as well as kneading of the boiled potatoes with red chilies, green chilies, coriander, turmeric powder, salt and dried seeds of pomegranate (dried seeds). The prepared paste was given circle shapes cutlets.
Eggs Cooking oil for frying	2 no 1/2 kilo	2. Then eggs were beaten and prepared potato cutlets were fried at 180°C temperature for 3 min (T_1_) and 4 min (T_2_) each.

**TABLE 8:** Ingredients and procedure for preparation of chicken nuggets.

Ingredients	Measures	Procedure
Boneless chicken Soy sauce Salt White chillies Garlic paste Black pepper	1 kilo 2 Tbsp Few points 1 tsp 1 tsp 1/2 tsp	1. Firstly, chicken was marinated with salt, soya sauce, white chilies, garlic, and black peppers. Then this material was refrigerated for 1 to 2 h.
Egg white White flour (maida) Corn flour Salt White cumin seeds	2 no 1 cup 1/2 cup Few points 1 tsp	2. Egg white was beated and mixed with flour, corn flour, and salt until paste was developed. Then chicken pieces were dipped into the past and refrigerated them for 15 to 20 min.
All purpose flour Salt	1/2 kilo Few pints	3. After this these chicken pieces were fried till appearance of golden color. In this way, nuggets were prepared. These nuggets were fried at 180°C temperature for 3 min (T_1_) and 4 min (T_2_) each.

**TABLE 9:** Treatment plan of different bakery and snack/fried products prepared under various time and temperature conditions.

Bakery products		
Products	Temperature of baking	Time of baking
Pizza	190°C (T_1_)	20 min
	210°C (T_2_)	
Cakes	170°C (T_1_)	20 min
	190°C (T_2_)	
Muffins	170°C (T_1_)	20 min
	190°C (T_2_)	
Biscuits	170°C (T_1_)	20 min
	190°C (T_2_)	

**Snack/fried products**		

**Products**	**Temperature of frying**	**Time of frying**

Samosas	180°C	4 min (T_1_)
		5 min (T_2_)
Pratha rolls	180°C	4 min (T_1_)
		5 min (T_2_)
Chicken nuggets	180°C	3 min T_1_)
		4 min (T_2_)
Potato cutlets	180°C	3 min (T_1_)
		4 min (T_2_)

### Chemical analysis of flour

First of all, straight-grade flour was assessed for proximate composition, that is, moisture, crude protein, crude fat, crude fiber, and ash contents. Moisture contents of flour were examined using an air-forced draft oven at a temperature of 105°C, by following the method no. 44-15A described in AACC International (25). Ash contents of flour were examined using a muffle furnace at 550°C for 6 h, by following the method no. 08-01 described in AACC International (25). The crude fat of flour was determined using the Soxhlet apparatus according to the method no. 30-25 described in AACC International (25). Crude fiber of flour was tested after fat extraction by following the method no. 32-10 described in AACC International (25). Crude protein contents of flour were determined using Kjeldahl’s apparatus by following method no. 46-10 as described in AACC International (25). The protein (%) was calculated by multiplying % nitrogen with a factor of 5.7. Wet and dry gluten contents of wheat flour were measured by using a Glutomatic system by following method no. 38-12 described in AACC International (25).

### Rheological properties of dough

Wheat flour was examined for physical dough properties by using Brabender Farinograph (C. W. Brabender, Duisburg, Germany) according to the method no. 54-21 described in AACC International (25) at Ayub Agriculture Research Institute, Faisalabad. The farinograms were interpreted for different characteristics such as water absorption, dough development time, dough stability, mixing tolerance index, and softening of the dough. The instrument automatically determines the amount of flour to be poured into the mixer of the farinograph based on the moisture content of the flour. The farinograph is equipped with a 300 g capacity mixer. Mixing was carried out for 20 min. When the mixing started, immediately the computer automatically started to plot the graph.

### α-amylase activity (falling number) of wheat flour

The α-amylase activity of wheat flour was studied through the falling number at Ayub Agriculture Research Institute, Faisalabad. The falling number of flour was recorded with the help of Falling Number Apparatus 1,900 (Perten instruments AB, SE 14105, Huddinge, Sweden) according to the method no. 56-81 described in AACC International (25). About 25 mL of water was added to 7 g of flour in the falling number tubes. A rubber stopper was inserted and tubes were shaken in an upright position 30 times up and down until mixed. The developed slurry was heated in a water bath at 80°C and stirred constantly so that the starch gelatinized and formed a thick paste. The tubes were inserted into the falling number machine and the time taken by the stirrer to drop through the flour paste was recorded as the falling number value.

### Extraction and quantification of acrylamide

After product development, all the samples were analyzed for their acrylamide contents through the HPLC method in the Laboratory of Food Technology department at Pakistan Council of Scientific and Industrial Research (PCSIR), Lahore, using the method described by Khoshnam et al. (26).

About 10 g of crushed and homogenized sample was mixed with 25 mL of n-hexane in a flask. The flask was placed in a shaker for 20 min. This step was repeated three times for thorough de-fattening of the sample by decantation of hexane. The sample was dried using a hot plate at a moderate temperature (70–80°C). For extraction of acrylamide, acetone (50 mL) and distilled water (100 mL) were added into the sample in a flask and placed in a water bath at 40°C for 20 min. The acetone was filtered and further evaporated by putting it on a hot plate again. The residues were suspended in 5 mL of distilled water and filtered with a 0.25 micron syringe filter to run on HPLC. The stock solution of standard acrylamide (2,000 ppb) was prepared by using HPLC-grade distilled water and different dilutions were prepared from the stock solution to construct a calibration graph for its quantification in samples.

Samples were analyzed using the HPLC Perkin-Elmer 200 Series (USA), with C-18 Column [Agilent 5 TC-C 18 (2) 250 length × 4.6 mm diameter] and with a porosity of 5 microns (made in the Netherlands). The column oven temperature was set at 40°C and the flow rate of the mobile phase was maintained at 1 mL/min. The analysis was performed using a 20 μl injection loop and a UV detector adjusted at 202 nm.

### Sensory evaluation

A voluntary panel of 10 judges consisting of males and females was selected from the Government College University, Faisalabad. Panelists were educated on testing terminologies and requested to evaluate the various food samples prepared in the present study using a nine-points hedonic scale. Pizza, cake, and muffins were evaluated for color, taste, odor, texture, and overall acceptability according to Gladys et al. (27). Biscuits were evaluated for color, taste, crispness, texture, and overall acceptability according to Al-Marazeeq and Angor (28). Chicken nuggets and potato cutlets were evaluated for color, flavor, tenderness, juiciness, and overall acceptability according to Kim et al. (29). Sensory evaluation (color, aroma, texture, taste, and overall acceptability) of parathas and samosa were carried out according to Rosli et al. (30).

### Statistical analysis of data

The results obtained in the present study were then analyzed statistically using a paired *t*-test (for comparison between T_1_ and T_2_) through Minitab software to determine the significance level (*p* < 0.05). The data for acrylamide concentration and sensory characteristics were analyzed through this technique.

## Results and discussion

### Proximate analysis of raw material

The results obtained for proximate analysis of patent flour to be used for the preparation of different products in the present study are shown in Table 10.

**TABLE 10:** Proximate composition of wheat flour purchased from Faisalabad market.

Contents	Quantity (%)
Ash	1.07 ± 0.03
Crude protein	10.65 ± 0.4
Moisture	11.02 ± 0.7
Crude fat	1.03 ± 0.02
Crude fiber	0.12 ± 0.01
Dry gluten	9.58 ± 0.40
Wet gluten	27.89 ± 1.11

In the present study, dry and wet gluten were 9.58 and 27.89%, respectively. Gluten is the protein of wheat. The flour from different wheat varieties in Pakistan showed wet and dry gluten (%) in the ranges 20.14 to 32.67 (%) and 7.27 to 10.68 (%), respectively (31, 32). Our results concerning wet and dry gluten (%) also fall in the above-mentioned ranges. The springiness of dough is attributed to the gluten content. Moreover, it also plays a vital role in the development of the texture of pizza and the firmness of the baked products due to its elastic nature (33).

It was observed that the flour purchased from the Faisalabad market contained 1.07% ash contents, 10.65% crude proteins, 11.2% moisture, 1.03% crude fat, and 0.12% crude fiber contents. Our results concerning ash, crude proteins, and moisture (%) contents of flour were consistent with the findings of Naseem et al. (31) and Iqbal et al. (32). Usually, the crude fiber and fat contents of patent flour are lower than whole wheat flour. As patent flour was used in the current study, the fiber contents were very low.

### Rheological properties of dough

The obtained results of farinographic studies are presented in Table 11. Rheological quality characteristics of flour influence other attributes, especially the sensory quality of bakery products. The results of the farinograph are used as parameters in formulation to estimate the amount of water required to make the dough, evaluate the effects of ingredients on mixing properties, evaluate flour blending requirements, and check flour uniformity. Moreover, the results are also used to predict processing effects, including mixing requirements for dough development, tolerance to over-mixing, and dough consistency during production. Farinograph results are also useful for predicting finished product texture characteristics. For example, strong dough mixing properties are related to firm product texture.

**TABLE 11:** Mean values for farinographic studies of wheat flour purchased from Faisalabad market.

Traits	Value
Water absorption	52.42 ± 1.67(%)
Dough stability	11.23 ± 0.79*min*
Dough development time	6.13 ± 0.34*min*
Arrival time	1.9 ± 0.11*min*
Departure time	11.47 ± 1.54*min*

Farinograph is used to determine the quality of the end product. The parameters of the Farinograph usually have a noteworthy role in the estimation of the amount of water for dough making, to evaluate flour blending requirements, to assess the effects of ingredients during mixing, and also to ensure flour uniformity (34).

It was observed that the mean value for water absorption was 52.42%, whereas dough stability and dough development time were 11.23 and 6.13 min, respectively. The arrival time was 1.9 min whereas the departure time was recorded as 11.47 min. Tehseen et al. (34) studied the rheological characteristics of whole wheat flour from different varieties to investigate the quality characteristics of pizza prepared from such flour. They observed water absorption of dough is in the range of 54.40 to 63.60 (%) which was higher than that observed in the present study because patent flour was used for making dough in our study. Strong gluten flour has a higher water absorption capacity than weak gluten flour. Similarly, dough stability and dough development times of whole wheat flour from different varieties were reported in the range of 3.90 to 13.20 min and 2.3 to 6.5 min, respectively (34, 35). Our results regarding dough stability and dough development times also lay in the above-reported ranges. Strong gluten has dough stability greater than 11 min (34). Moreover, arrival and departure times of whole wheat flour from different varieties were reported by Tehseen et al. (34) in the range of 1.40 to 2.90 min and 6.8 to 19.9 min, respectively. Our results regarding arrival and departure times also lay in the above-reported ranges.

### α-amylase activity (falling number) of wheat flour

The falling number of flour shows whether there is an alpha-amylase activity in the flour/grains or not. A high falling number indicates less sprout damage of wheat by amylase activity and vice versa. Therefore, the quality of flour should be checked before preparing any product. The quality of flour regarding the falling number test was optimum in the present study. The falling number of patent flour used for making different products in the present study was 342 (s) which falls in the range of 291.00 to 382.67, as reported by Naseem et al. (31). The falling number indirectly measures the amount of α-amylase activity in wheat. The activity of this enzyme increases as wheat germinates depending on weather conditions. The higher level of α-amylase activity in wheat flour shows a low falling number. The falling number of flour having values below 200 s do not consider good flour whereas falling number values exceeding 400 s indicate very low or no α-amylase activity (31).

### Acrylamide contents in products

**FIGURE 1:**
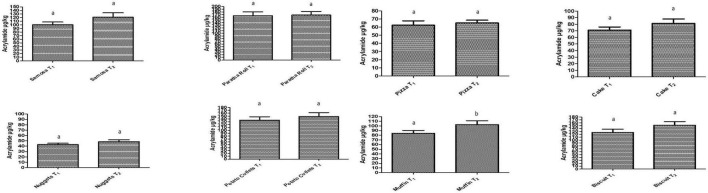
Acrylamide concentrations (μg/kg) in various bakery (Pizza, cake, muffins, and biscuits), snack (Chicken nuggets and potato cutlets), and fried (paratha roll and samosa) products.

The formation of acrylamide was investigated in each product separately under different treatments (temperature and time of cooking). The acrylamide concentrations found in different products in the present study are presented in Figure 1. There were slight variations in the contents of acrylamide between T_1_ and T_2_ for all the products prepared in the present study. But there was a significant variation of acrylamide concentrations between T_1_ and T_2_ of Muffins prepared in the present study. This means temperature affected the formation of acrylamide during the processing of all the products. The effect of time and temperature on the formation of acrylamide was also confirmed by different studies (36–38). The variations in acrylamide concentrations in different products might be due to variations in recipe ingredients as reported by Stojanovska and Tomovska (39). Among the bakery products, acrylamide concentration in T_1_ of pizza was 62.42 μg/kg whereas 65.21 μg/kg was observed in T_2_. Fermentation is involved in the preparation of pizza. In a study conducted on pizza by González-Mulero et al. (40), it was found that acrylamide contents were in the range of 15 to 104 μg/kg which was concurrent to the findings of the present study. The acrylamide levels analyzed in pizza samples from Italy were in the range of 90 to 250 μg/kg (41) which were higher than the values obtained in the present study. Such kind of discrepancies in acrylamide contents might be due to variations in the source of raw material (especially flour) affecting its formation (42). European Food Safety Association (EFSA) reported set mean values of acrylamide in pizza as 24 μg/kg which were lower than the values observed in the samples studied in the present study.

Similar to the findings of pizza, T_1_ of cake showed 71.21 μg/kg while T_2_ treatment showed increased concentration up to 81.19 μg/kg. The T_1_ of Muffin was found to contain an acrylamide concentration of 84.24 μg/kg which abruptly increased up to 102.84 μg/kg in T_2_. The variations in acrylamide concentrations in both products might be due to slight variations in recipe ingredients as reported by Stojanovska and Tomovska (39). As both cake and muffins are prepared due to the action of baking powder, their category regarding the texture of bakery products is more or less similar. Moreover, their baking temperature is also the same. Therefore, it would be logical to compare the results of muffins with cake. González-Mulero et al. (40) found acrylamide concentration in the range of 15 to 138 μg/kg in sponge cake which was in agreement with the findings in the present study. EFSA reported the set values of acrylamide in cake as 66 μg/kg which were in accordance with the values observed in the samples studied in the present study. Both cakes and muffins contain more quantity of oils/fats than pizza, therefore, it might be assumed that baking temperature may affect acrylamide concentrations.

A similar trend was also observed in biscuits as T_1_ showed 126.52 μg/kg acrylamide contents whereas T_2_ had 151.52 μg/kg. Among the bakery products, the maximum concentration of acrylamide was found in biscuits. Schouten et al. (43) set temperature of oven at 175°C (18 min) for baking of biscuits. They found acrylamide concentration in baked biscuits in the range of 130 to 154 μg/kg which was in line with the findings of the present study. EFSA reported 201 μg/kg of acrylamide as the mean value in biscuits which was above the values observed in the samples studied in the present study. The samples of biscuits were shown to have the maximum acrylamide content among bakery products investigated in the present study which might be due to the lower water activity of biscuits compared to other bakery products (44).

Among the fried products, the paratha roll showed the maximum acrylamide concentration. The treatment T_1_ of paratha roll was shown to have 165.92 μg/kg acrylamide concentration which slightly (non-significantly) increased in T_2_ (168.24 μg/kg). The treatment T_1_ of samosa contained 100.43 μg/kg acrylamide contents and T_2_ showed 121.29 μg/kg. Both paratha roll and samosa are traditionally used in the culture of Pakistan at fast food service chains. To the best of our knowledge, no literature was found concerning the investigation of acrylamide in paratha rolls and samosa. As potatoes were used in the preparation of these products, so it might be assumed that the contents of acrylamide in these products might be due to the processing of potatoes. The potato is usually rich in asparagines which participated in the Maillard reaction during processing and which might be the reason for the production of acrylamide in potatoes.

The treatment T_2_ (148.21 μg/kg) of potato cutlets contained higher acrylamide concentration than the T_1_ (135.71 μg/kg) treatment. The potato cutlets are also used traditionally by fast food services and domestic levels. No literature was found regarding the investigation of acrylamide concentrations in potato cutlets. It would be logical to compare the results of potato cutlets with potato-based products concerning acrylamide contents. It was investigated that acrylamide contents were in the range of 125 to 375 μg/kg in potato chips that were deep-fried (45). The potato cutlets prepared in the present study were shallow fried. Therefore, little bit lower contents of acrylamide were detected in the present study compared to the above-mentioned study. EFSA reported set values of acrylamide in fried potatoes as 201 μg/kg. As potato cutlets were also prepared from potatoes after frying, it may be assumed that the results would be correlated to fried potatoes. Our results of acrylamide levels in potato cutlets were slightly lower than that reported in EFSA. It has been reported that acrylamide concentration increased by increasing the temperature and time of frying of potato chips (46).

The minimum acrylamide concentration was observed in chicken nuggets. The treatment T_1_ of chicken nuggets was shown to have 43.04 μg/kg acrylamide concentration which slightly (non-significantly) increased in T_2_ (48.27 μg/kg). In a study conducted in Iran, it was observed that acrylamide was present in the range of 13 to 22 μg/kg in fried chicken nuggets which were more or less half as detected in the present study (37). Such discrepancies might be due to variations in processing conditions and raw materials used. Chicken nuggets contain chicken as basic raw material in their preparation. In another study, the levels of acrylamide were observed below 7 μg/kg in deep-fat dried chicken meat, which were also lower than that observed in the present study (47). Such discrepancies might be due to variations in processing conditions and raw materials used.

### Sensory evaluation of products

**FIGURE 2:**
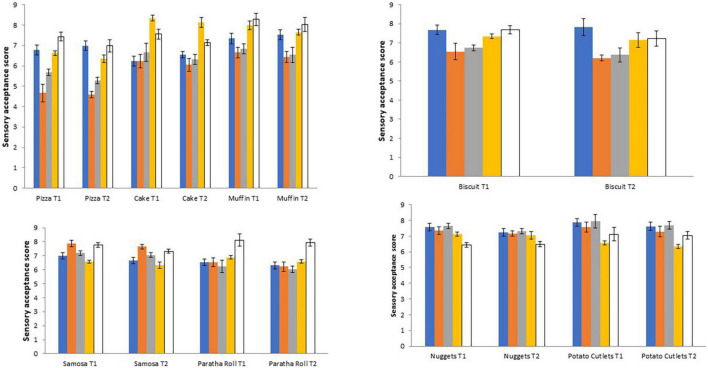
Sensory evaluation of various bakery (Pizza, cake, and muffins were evaluated for color, taste, odor, texture, and overall acceptability; Biscuits were evaluated for color, taste, crispness, texture, and overall acceptability), snack (Chicken nuggets and potato cutlets were evaluated for color, flavor, tenderness, juiciness, and overall acceptability), and fried (parathas and samosa of color, aroma, texture, taste, and overall acceptability) products.

The results concerning the sensory evaluation of products investigated in the present study are presented in Figure 2. Sensory evaluation was carried out to check the acceptability of prepared products. There might be possibilities that poor sensory evaluation may be due to over-frying or over-baking at high temperatures, thereby leading to more acrylamide contents. All the investigated products were checked sensorially to check optimum baking and frying conditions and the products with good overall acceptability were selected for investigation of acrylamide contents. The bakery products, that is, pizza, cake, and muffins were evaluated for color, taste, odor, texture, and overall acceptability. All these attributes of sensory evaluation of the aforementioned products slightly decreased by increasing the temperature (as in treatment T_2_). Among these bakery products, the highest score regarding color was obtained by muffins (T_1_ = 7.31, T_2_ = 7.52) whereas the least score was given to pizza. Similarly, regarding taste, the lowest score was obtained by pizza (T_1_ = 4.67, T_2_ = 4.58) and the highest score was given to muffins (T_1_ = 6.66, T_2_ = 6.43). A similar trend was shown regarding the odor of these products. Regarding texture, the highest score was obtained by cake (T_1_ = 8.34, T_2_ = 8.13) whereas pizza obtained the lowest score (T_1_ = 6.62, T_2_ = 6.34). Concerning overall acceptability, the highest score was obtained by muffins followed by cake. The sensory attributes (color, taste, crispness, texture, and overall acceptability) of biscuits were different compared to the above-mentioned bakery products. All the sensory attributes showed slightly lower scores in treatment T_2_.

The fried products, that is, samosa and paratha rolls, were evaluated for color, aroma, taste, texture, and overall acceptability. All these attributes of sensory evaluation of the aforementioned products slightly decreased by increasing the temperature as in treatment T_2_. The highest score regarding color was obtained by samosa (T_1_ = 6.98, T_2_ = 6.66) whereas lower score was given to paratha roll. Similarly, regarding aroma, lower score was obtained by the paratha roll (T_1_ = 6.53, T_2_ = 6.22) and higher score was given to samosa (T_1_ = 7.87, T_2_ = 7.64). A similar trend was shown regarding the taste of these products. Regarding texture, higher score was obtained by paratha roll (T_1_ = 6.88, T_2_ = 6.59) whereas samosa obtained lower score (T_1_ = 6.57, T_2_ = 6.32). Concerning overall acceptability, the highest score was obtained by paratha roll.

The sensory attributes (color, flavor, tenderness, juiciness, and overall acceptability) of chicken nuggets and potato cutlets were different compared to the above-mentioned fried products. Both of these products are shallow fried. All these attributes of sensory evaluation of the aforementioned products slightly decreased by increasing the temperature as in treatment T_2_. The highest score regarding color was obtained by potato cutlets (T_1_ = 7.88, T_2_ = 7.62) whereas a lower score was given to chicken nuggets. Similarly, regarding flavor, lower score was obtained by chicken nuggets (T_1_ = 7.34, T_2_ = 7.16) and higher score was given to potato cutlets (T_1_ = 7.56, T_2_ = 7.28). Regarding tenderness, lower score was obtained by chicken nuggets (T_1_ = 7.65, T_2_ = 7.32) and higher score was given to potato cutlets (T_1_ = 7.94, T_2_ = 7.68). A similar trend was shown regarding the juiciness of these products. Concerning overall acceptability, the highest score was obtained by potato cutlets.

## Conclusion

Among all the investigated products, the highest concentrations of acrylamide were observed in paratha roll, (165.92 μg/kg) followed by biscuits (126.52 μg/kg), and samosa (100.43 μg/kg). There were slight variations in the contents of acrylamide between T_1_ and T_2_ for all the products prepared in the present study. But there was a significant variation of acrylamide concentrations between T_1_ and T_2_ of Muffins prepared in the present study. Thus, it may be concluded that different time and temperature conditions resulted in varying concentrations of acrylamide compounds in different bakery and fried products. Moreover, baking or frying at higher temperatures produced more acrylamide concentrations. Therefore, it is suggested that the products investigated in the present study should be consumed every now and then and not on a routine basis to avoid the deleterious effect of acrylamide on our health.

## Data availability statement

The raw data supporting the conclusions of this article will be made available by the authors, without undue reservation.

## Author contributions

MA: designed and planned research work and carried out research in the laboratory and helped in writing the manuscript. TQ: analyzed the whole data statistically and helped in writing the manuscript. MM: helped in organizing data. AA and UM: helped in reviewing and writing the manuscript. SI: helped in data analysis, funding acquisition, and reviewing the manuscript. AR: helped in conducting the research, analysis, and organizing the data. MI, SS, and MS: helped in revising data analysis and reviewing and finalizing the manuscript. TI: helped in analysis and organizing the data. AJ: helped in conducting the research and analysis. SS: helped in analysis. All authors contributed to the article and approved the submitted version.
