# Supplementary material for: Influence of baking and frying conditions on acrylamide formation in various prepared bakery, snack, and fried products
Source: Front Nutr. 2022 Nov 21;9:1011384. doi: 10.3389/fnut.2022.1011384 (PMC9749820; doi:10.3389/fnut.2022.1011384)
Supplement: Supplementary file 1 [file Data_Sheet_1.docx]

**Figure 1.** Chromatograms of Bakery products showing acrylamide peaks.

 
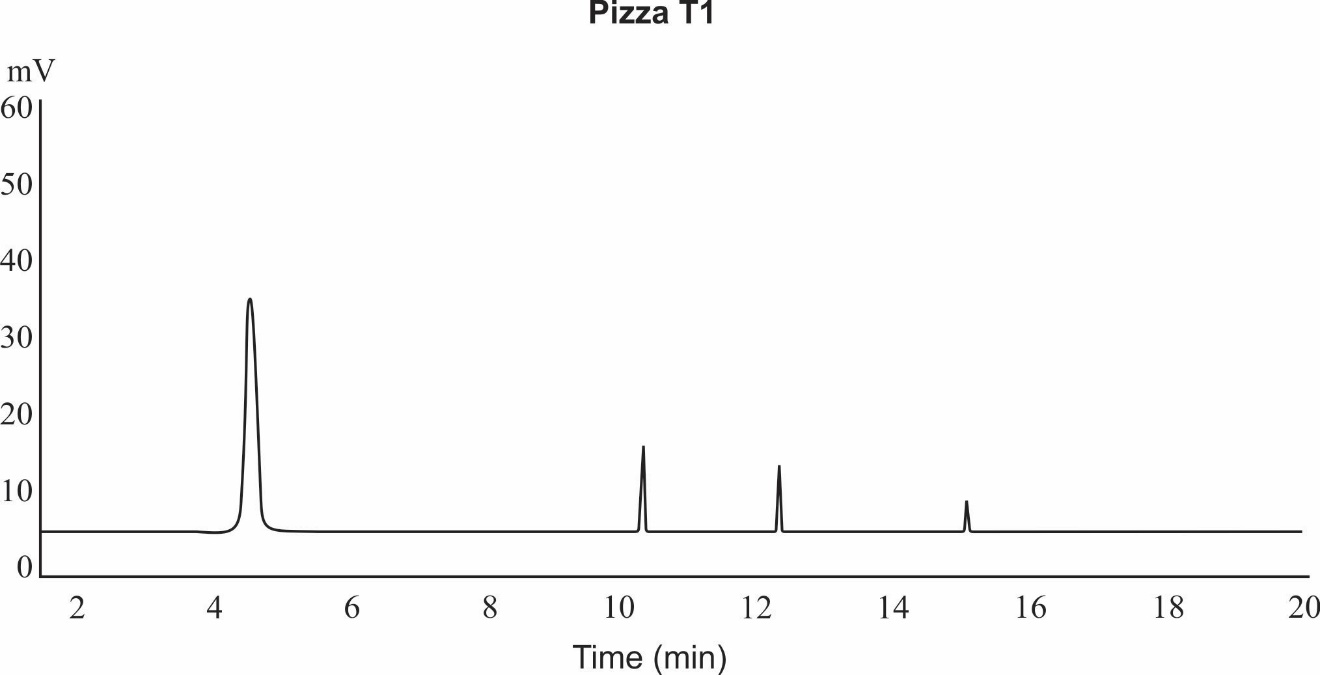

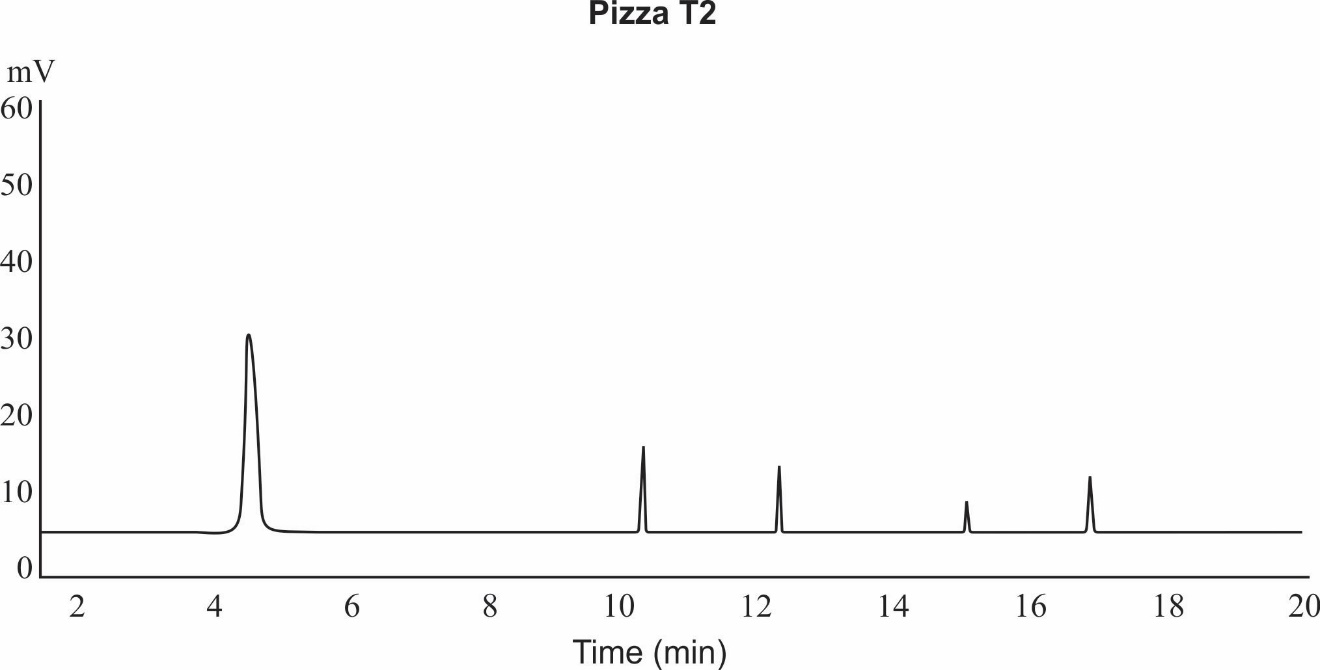

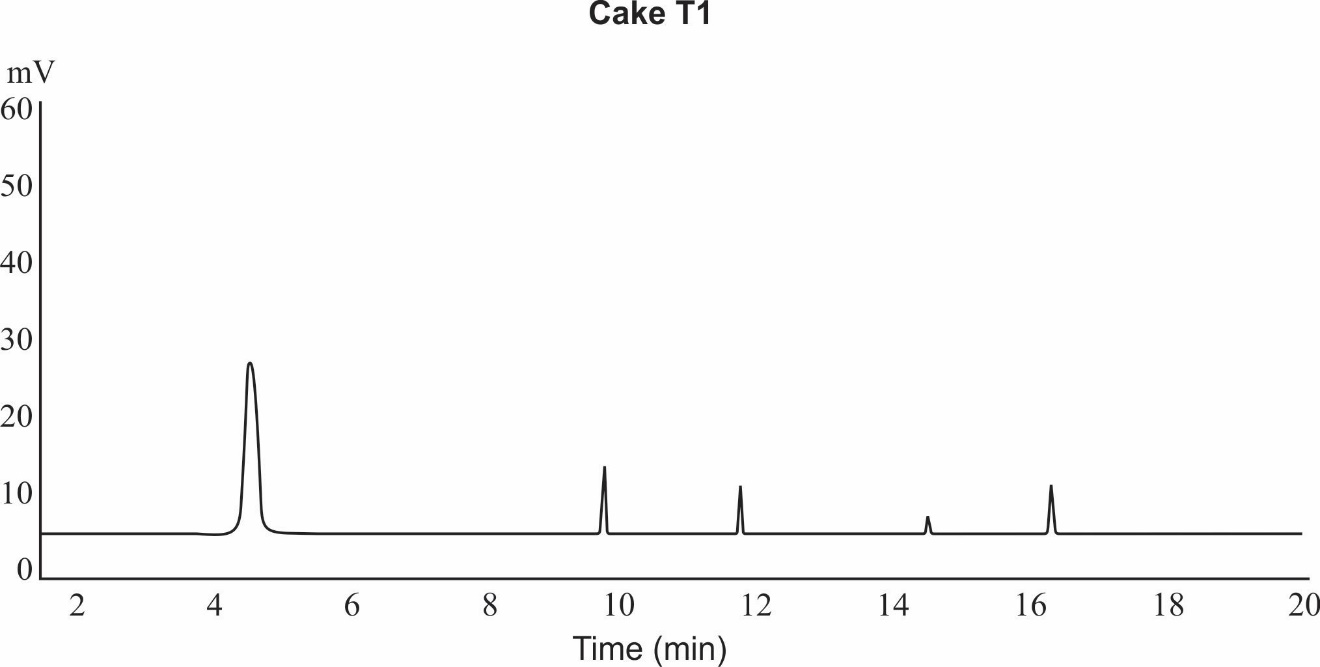

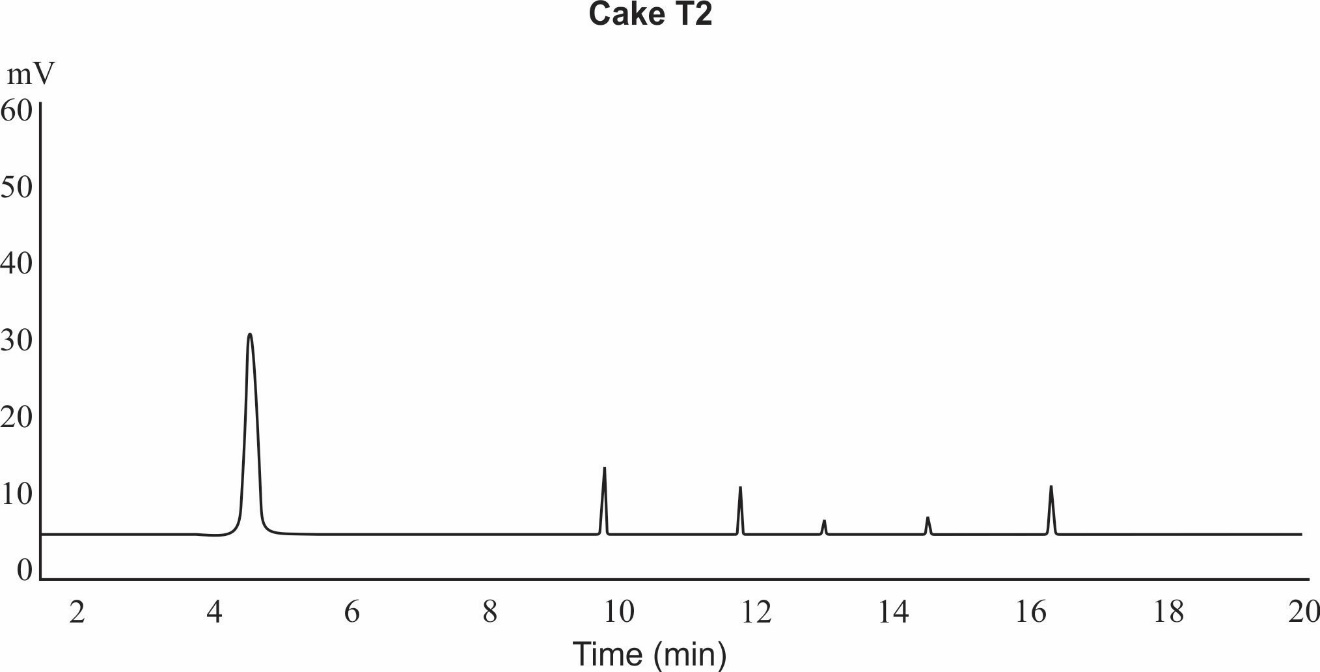


Acrylamide peak

Acrylamide peak

Acrylamide peak

Acrylamide peak

**Figure 2.** Chromatograms of Bakery products showing acrylamide peaks.

 
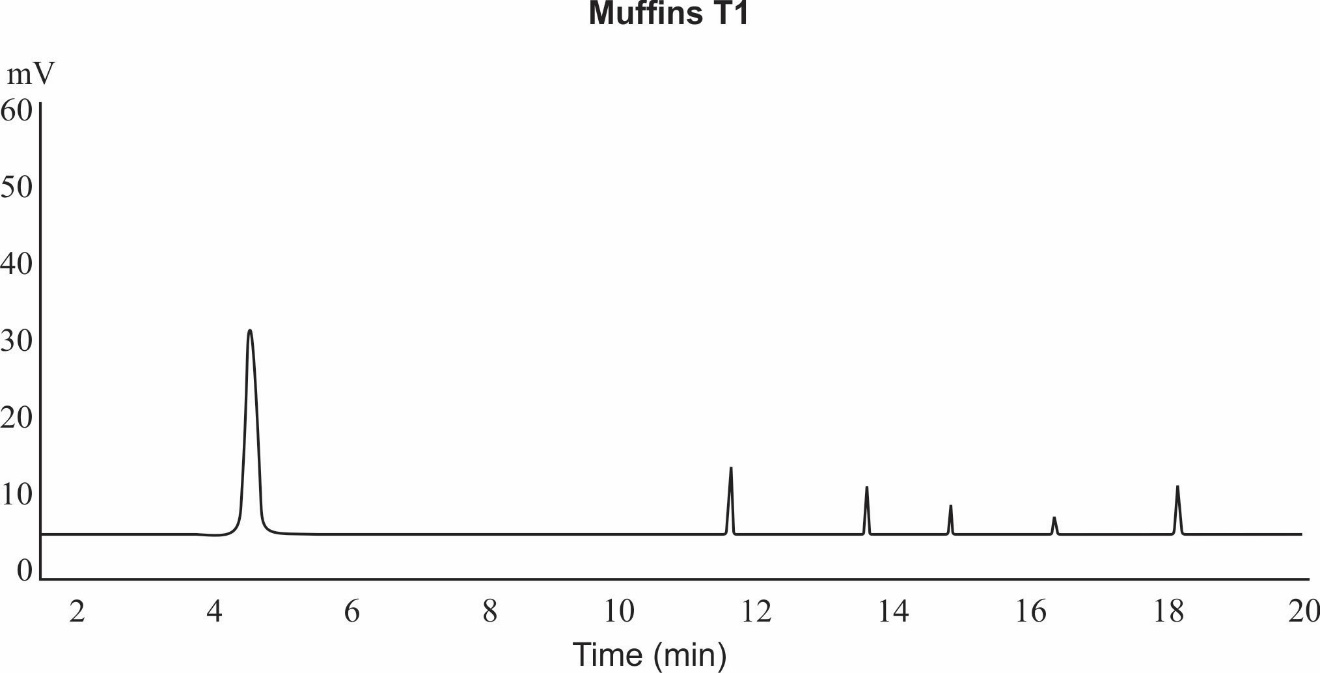

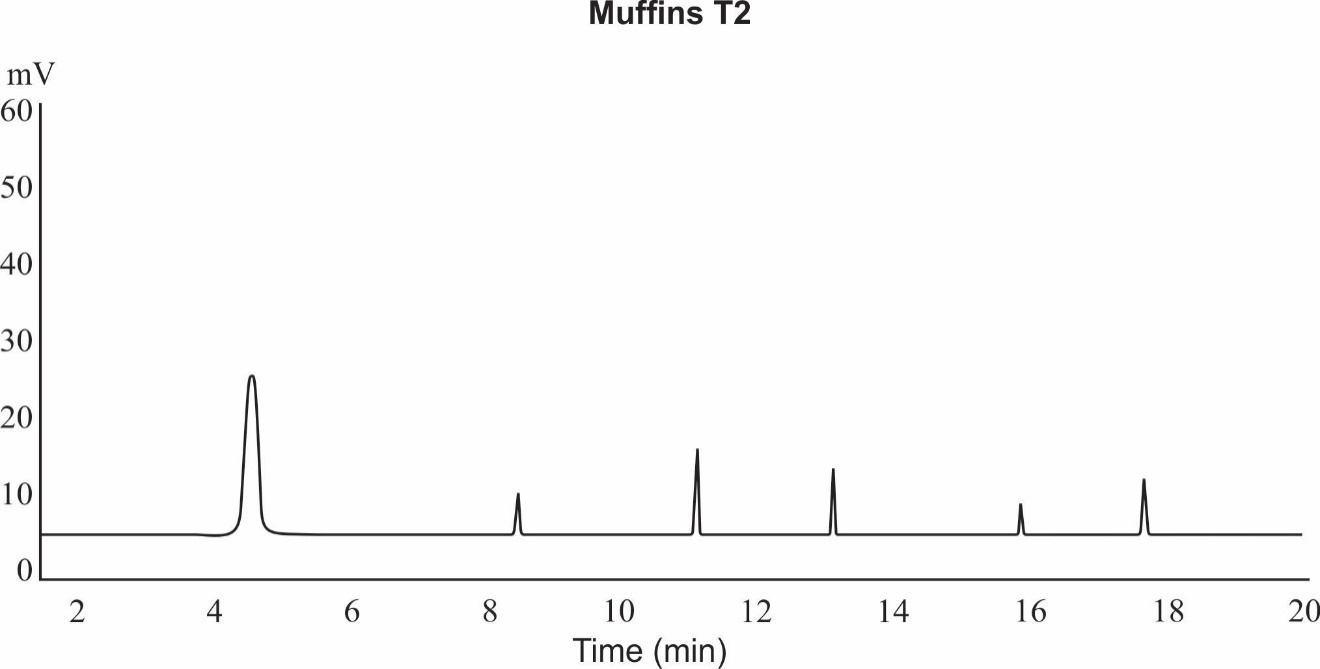

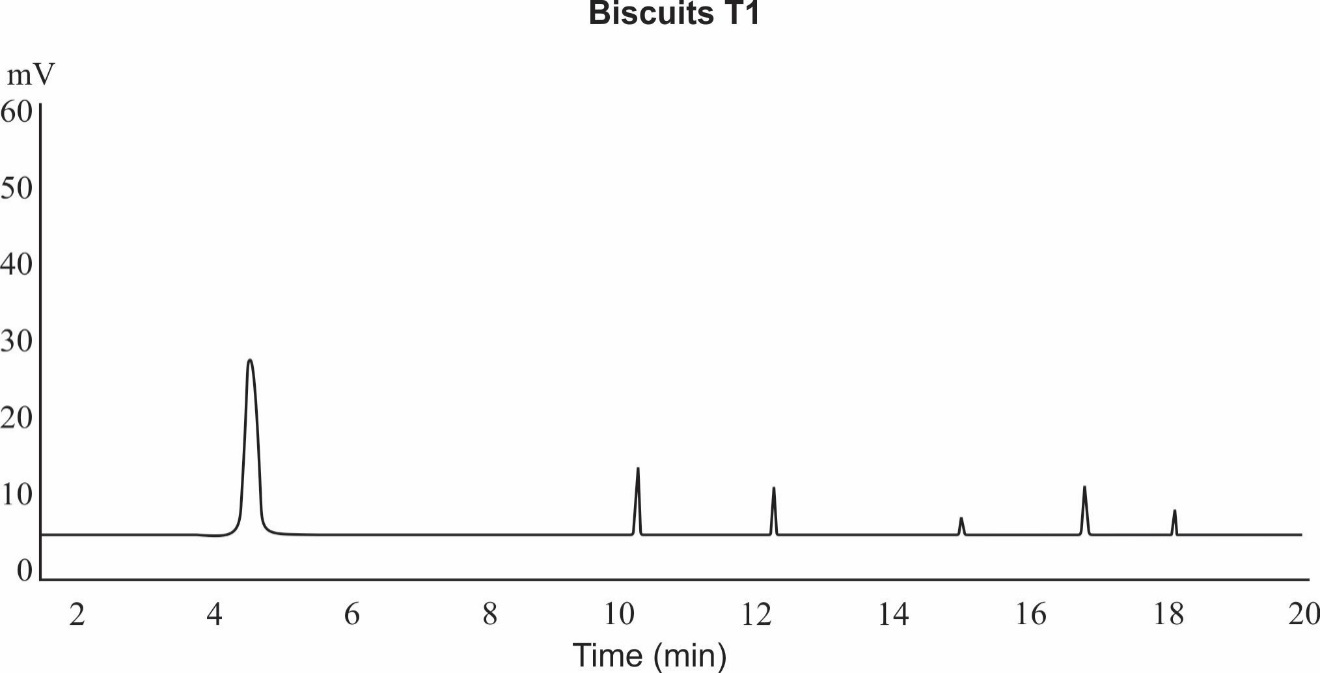

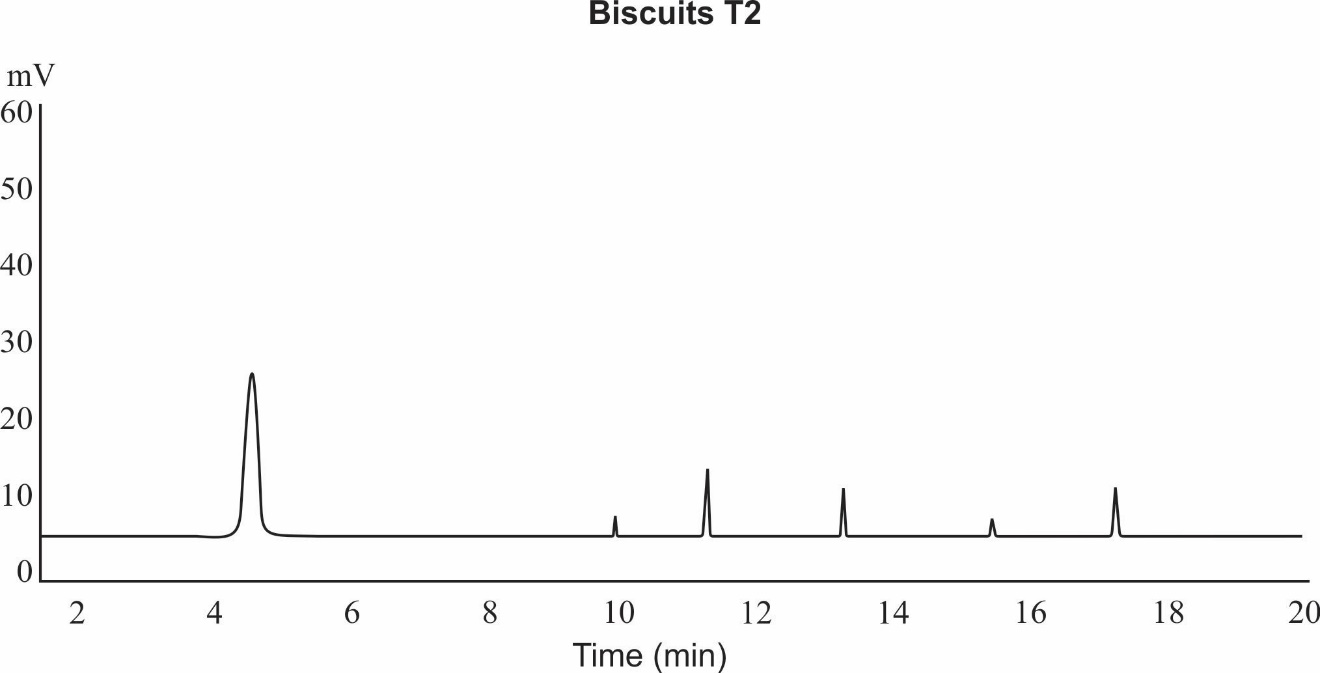


Acrylamide peak

Acrylamide peak

Acrylamide peak

Acrylamide peak

**Figure 3.** Chromatograms of Fried products showing acrylamide peaks.


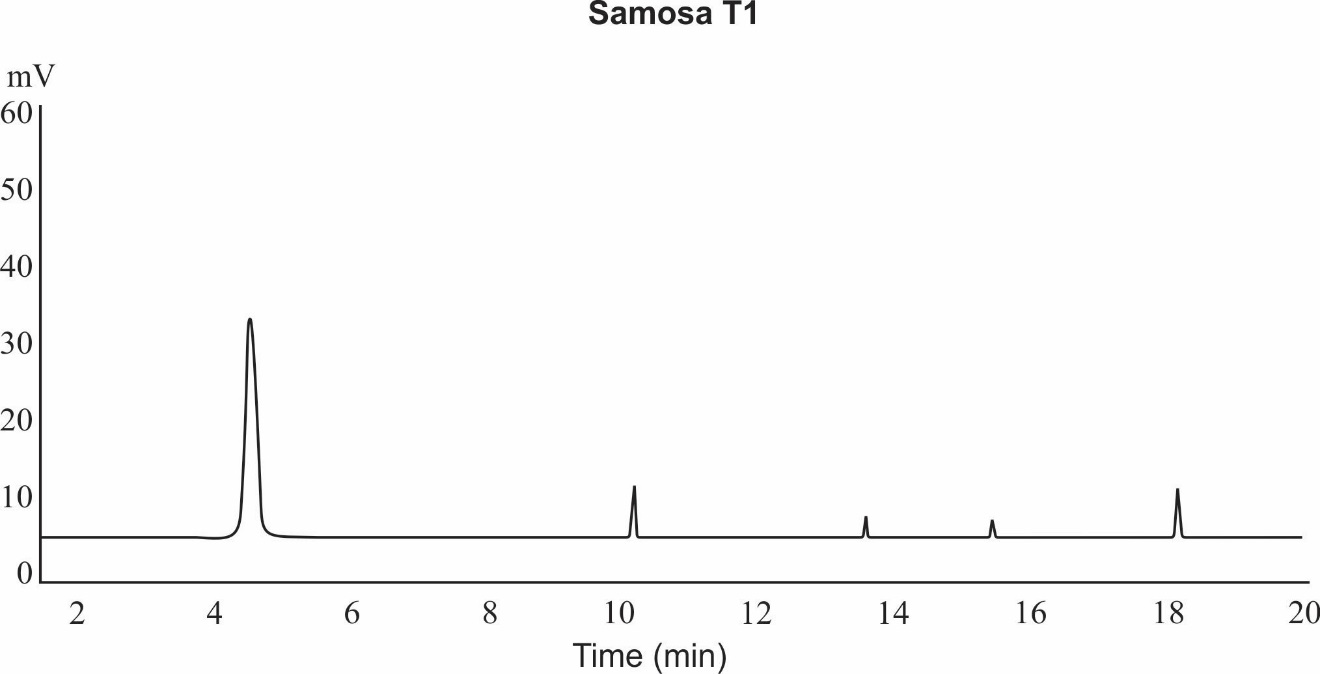

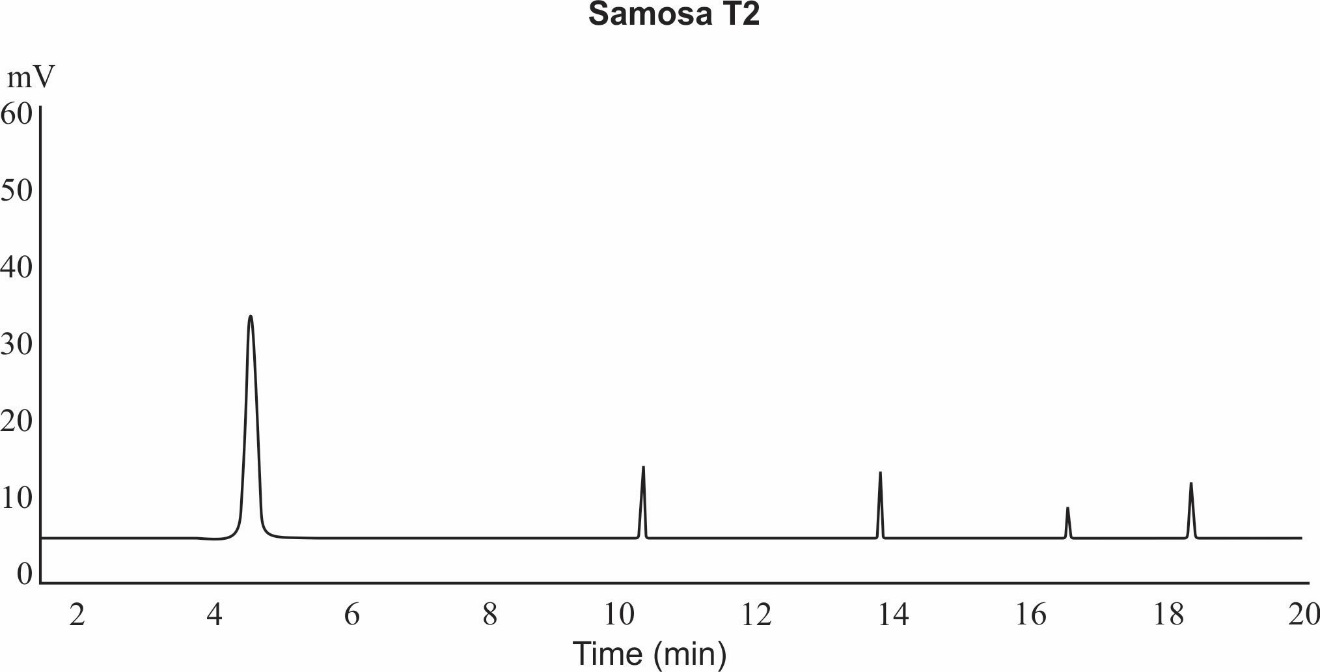


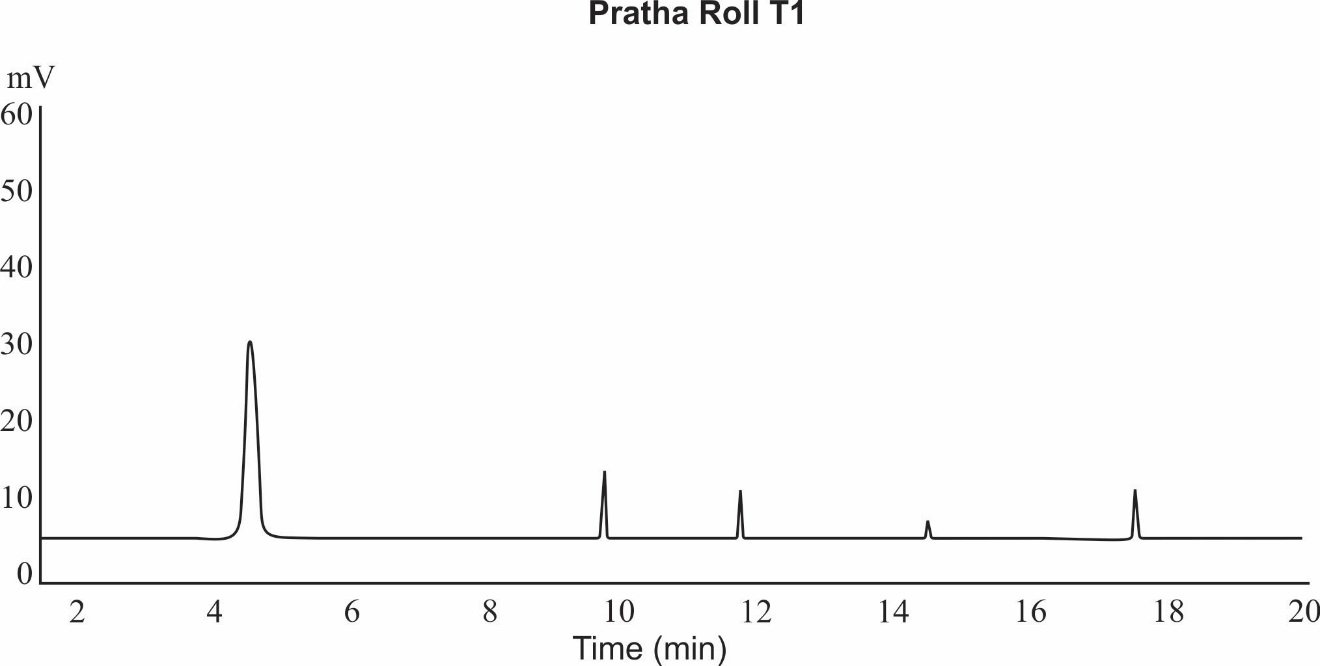

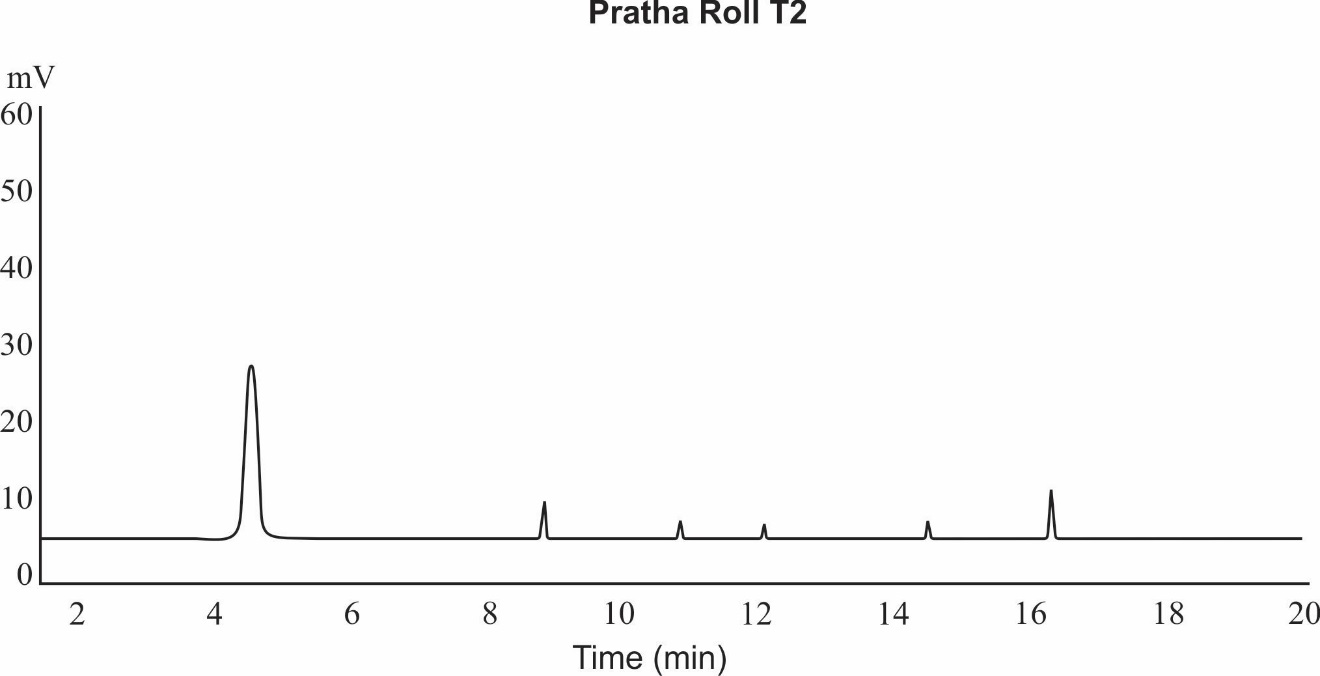


Acrylamide peak

Acrylamide peak

Acrylamide peak

Acrylamide peak

**Figure 4.** Chromatograms of Snack products showing acrylamide peaks.


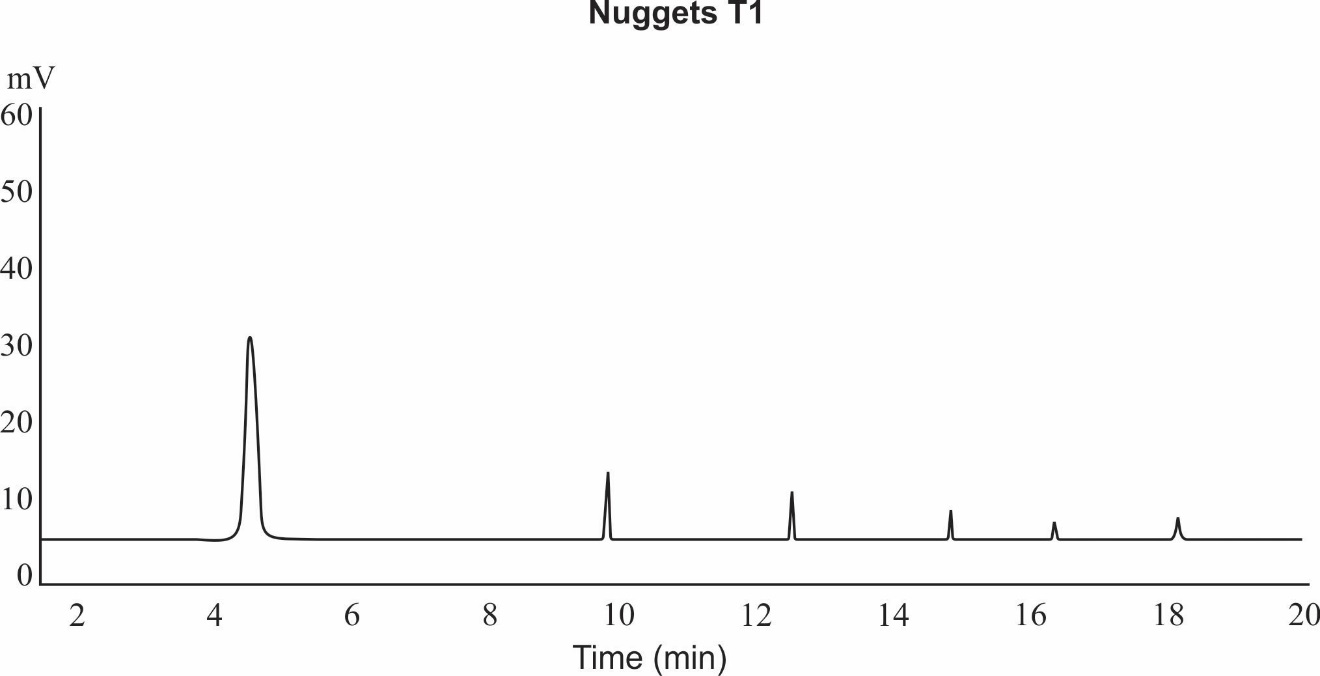

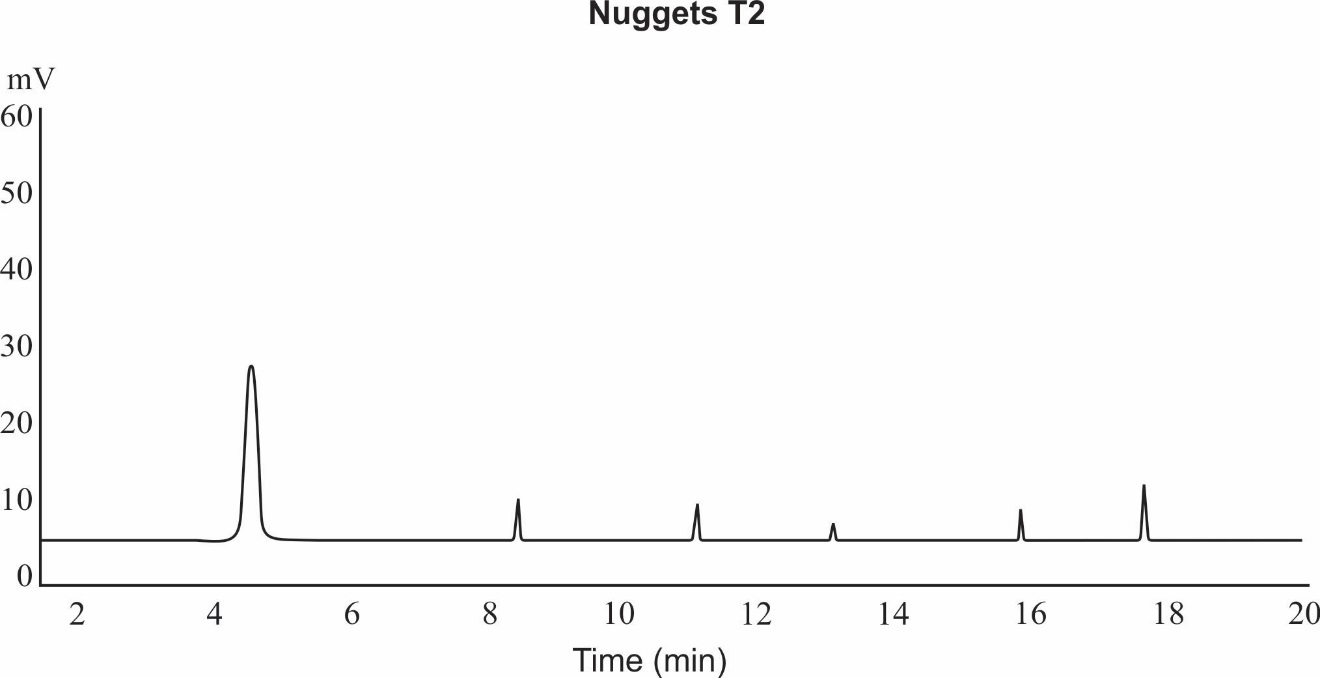

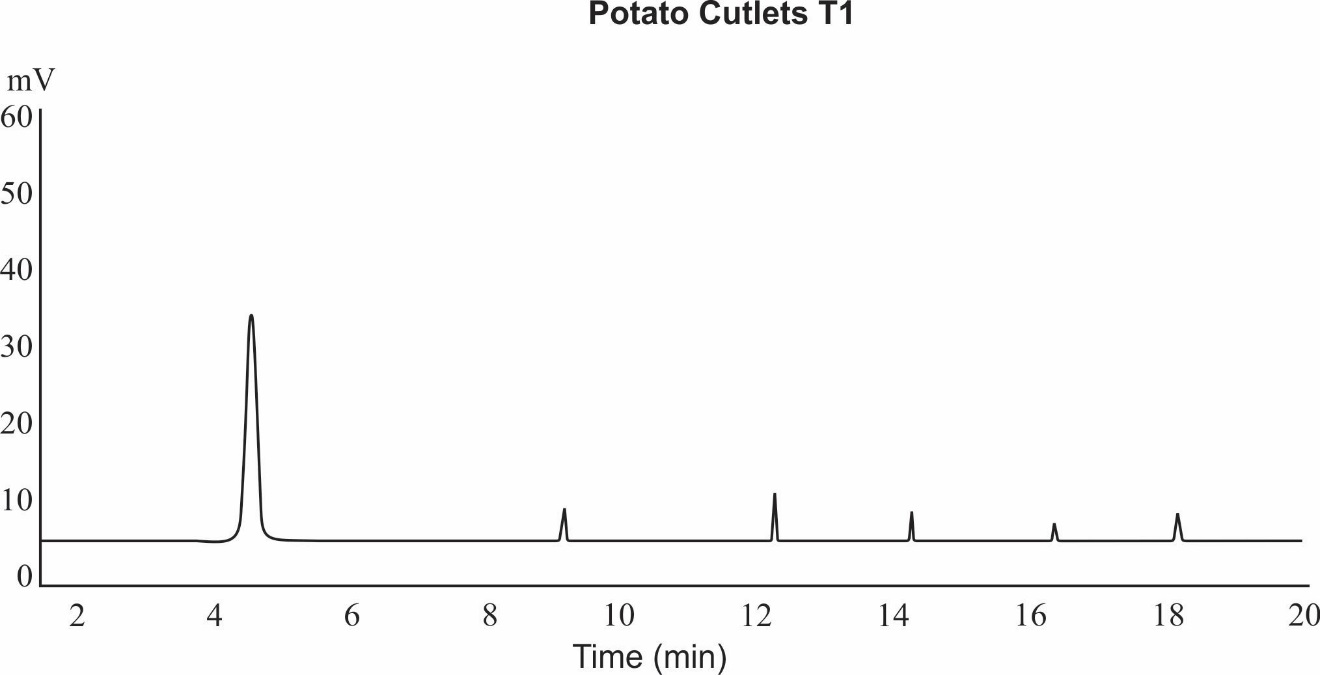

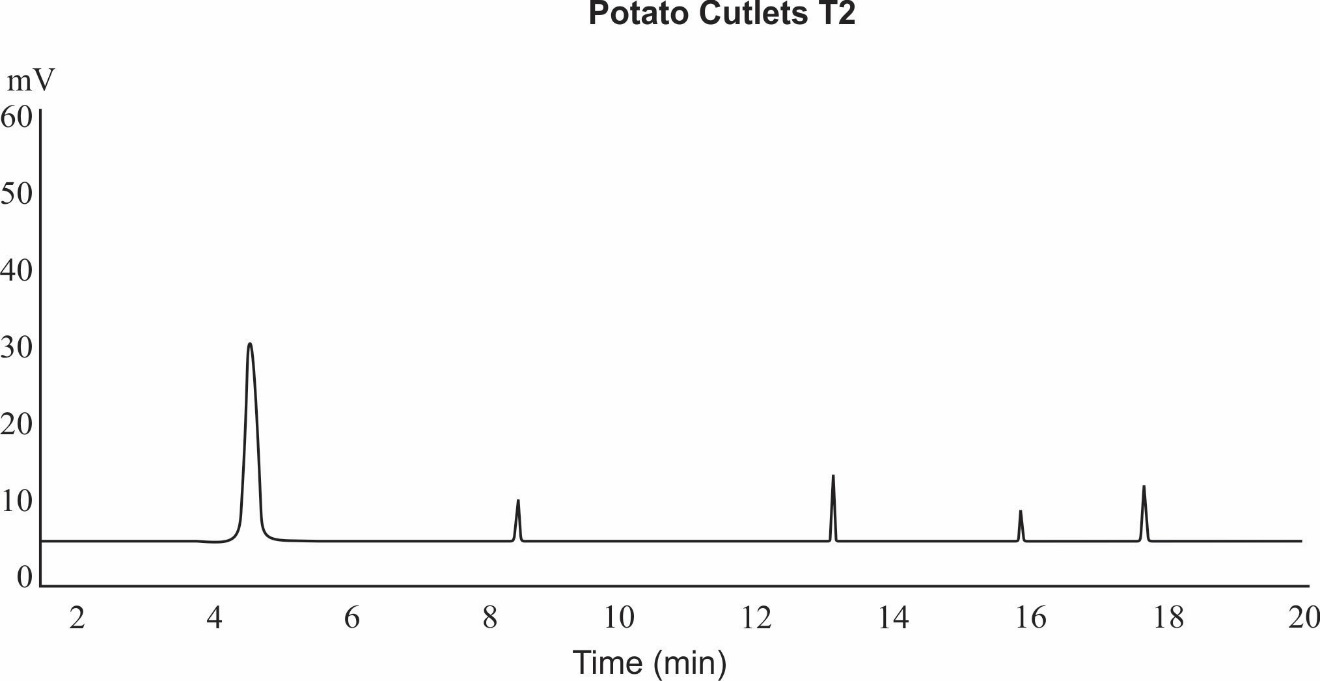


Acrylamide peak

Acrylamide peak

Acrylamide peak

Acrylamide peak
